# CCL25-CCR9 interaction modulates ovarian cancer cell migration, metalloproteinase expression, and invasion

**DOI:** 10.1186/1477-7819-8-62

**Published:** 2010-07-22

**Authors:** Erica L Johnson, Rajesh Singh, Shailesh Singh, Crystal M Johnson-Holiday, William E Grizzle, Edward E Partridge, James W Lillard

**Affiliations:** 1 Department of Microbiology, Biochemistry, & Immunology, Morehouse School of Medicine, 720 Westview Drive SW, Atlanta, GA 30310-1495, USA; 2 Department of Pathology, University of Alabama at Birmingham, 703 19th Street South, Birmingham, AL 35294-0007, USA; 3 Department of Obstetrics & Gynecology, Division of Gynecological Oncology, 618 20th Street South, University of Alabama at Birmingham, Birmingham, AL 35233-7333, USA

## Abstract

**Background:**

Ovarian carcinoma (OvCa) is the most lethal gynecological malignancy among women and its poor prognosis is mainly due to metastasis. Chemokine receptor CCR9 is primarily expressed by a small subset of immune cells and its only natural ligand, CCL25, is largely expressed in the thymus, which involutes with age. Other than the thymus, CCL25 is expressed by the small bowel. Interactions between CCL25 and CCR9 have been implicated in leukocyte trafficking to the small bowel, a frequent metastatic site for OvCa cells. The current study shows OvCa tissue and cells significantly express CCR9, which interacts with CCL25 to support carcinoma cell migration and invasion.

**Methods:**

RT-PCR and flow cytometry techniques were used to quantify the expression CCR9 by OvCa cells. OvCa tissue microarrays (TMA) was used to confirm CCR9 expression in clinical samples. The Aperio ScanScope scanning system was used to quantify immunohistochemical staining. Cell invasion and migration assays were performed using cell migration and matrigel invasion chambers. Matrix metalloproteinase (MMP) mRNAs were quantified by RT-PCR and active MMPs were quantified by ELISA.

**Results:**

Our results show significantly (*p *< 0.001) higher expression of CCR9 by mucinous adenocarcinoma, papillary serous carcinoma, and endometriod ovarian carcinoma cases, than compared to non-neoplastic ovarian tissue. Furthermore, CCR9 expression was significantly elevated in OvCa cell lines (OVCAR-3 and CAOV-3) in comparison to normal adult ovarian epithelial cell mRNA. OvCa cells showed higher migratory and invasive potential towards chemotactic gradients of CCL25, which was inhibited by anti-CCR9 antibodies. Expression of collagenases (MMP-1, -8, and -13), gelatinases (MMP-2 and -9), and stromelysins (MMP-3, -10, and -11) by OvCa cells were modulated by CCL25 in a CCR9-dependent fashion.

**Conclusions:**

These results demonstrate both biological significance and clinical relevance of CCL25 and CCR9 interactions in OvCa cell metastasis.

## Background

Ovarian Cancer (OvCa) is the fifth leading cause of cancer-related deaths among women in the United States [1,2]. OvCa has been viewed as an intraperitoneal disease that rarely spreads to other organs. However, recent autopsy studies revealed a much higher rate of occult metastasis, indicating extraperitoneal spread occurs with much greater frequency than previously appreciated and hematogenous dissemination of tumor cells occurs early and throughout all stages of OvCa [3]. For metastasis to occur, OvCa cells must disseminate from the primary tumor, penetrate the basement membrane and invade the interstitial stroma. Matrix metalloproteinases (MMPs) are structurally and functionally related zinc-dependent endopeptidases that normally function in ovulation, wound repair, and bone remodeling [4]. MMPs can be divided into three distinct categories based on their structural and functional properties: collagenases (MMP-1, -8, and -13), gelatinases (MMP-2 and -9), and stromelysins (MMP-3, -10, and -11). Collagenases initiate degradation of several naive fibrillar collagens. Gelatinases, also called type IV collagenases, degrade collagen and basement membrane components. Stromelysins can degrade a broad range of substrates, including collagen, fibronectin, laminin, elastin and proteoglycan core proteins [5]. High plasma and ascites fluid levels of MMPs have been correlated with OvCa progression and poor prognosis [6-8]. We have previously shown that CXCL12 and CCL25 can modulate the expression of MMPs by prostate cancer cells [9,10].

Chemokines represent a super-family of small, chemotactic cytokines that are involved in many inflammatory processes. Many cancer cell types display restricted expression of chemokine receptors [11,12]. CCR6 is overexpressed by liver metastases of ovarian carcinomas, suggesting CCL20-CCR6 interactions promote malignant cancer cells to metastasize to the liver [13]. It has also been shown that CXCL12 (stromal-derived factor, SDF-1α) affects the growth and metastasis of OvCa cells through interactions with CXCR4 [14]. Unfortunately, CXCR4 is not a tumor-specific marker and its ligand, CXCL12, is widely expressed by cells of the immune, cardiovascular, and nervous systems. Moreover, this chemokine plays an important role in fetal development, cardiovascular function, migration of hematopoietic stem cells and trafficking of naïve lymphocytes [11]. Deletion of either CXCR4 or CXCL12 is lethal to the embryo. Nonetheless, CXCL12-CXCR4 interactions enhanced intraperitoneal dissemination of OvCa cells [15], in part through activating MMP-2 and -9 [16].

CCL25 is mainly expressed by the thymus and small bowel as well as by the spleen after challenge with lipopolysaccharide [17,18]. Unlike CXCR4 and CXCL12, the deletion of either CCR9 or CCL25 genes is not lethal [19]. Hence, there may be fewer toxicities associated with therapies that target this axis. We show that CCR9 is expressed at higher levels by human OvCa cells and tissues in comparison to non-cancerous samples. Additionally, we show that CCL25 modulates MMP expression and enhances the migration and invasive potential of OvCa cells. These findings suggest CCL25-CCR9 interaction contribute to OvCa cell migration and invasion and blocking this axis might inhibit OvCa cell metastasis.

## Materials and methods

### OvCa tissue microarray

OvCa tissue microarrays were obtained from the Southern Division of the Cooperative Human Tissue Network (CHTN) and the University of Alabama at Birmingham. To construct these tissue microarrays, at least two cores (1 mm in diameter) per patient were arrayed on a receiver blank paraffin block. A qualified pathologist concerning the histopathology, the class and the grade of the tumor validated each core of the tissue microarray one additional time. The OvCa tissue microarray used in this study was composed of tumors from 34 patients. These ovarian tumors represented all histopathological subtypes (8 non-neoplastic, 10 serous adenocarcinoma, 11 endometrioid adenocarcinoma, 5 mucinous adenocarcinoma) and every tumor grade of OvCa disease. The tissue microarray was cut in 4 μm sections and placed on super frost charged glass microscope slides.

### Quantitation of immunohistochemical staining

To numerically analyze the immunohistochemical staining, virtual slides were created from stained samples after scanning each specimen using an Aperio ScanScope GL scanning system (Aperio Technologies). The ScanScope GL system generated true color digital images of each stained sample, which were viewed using ImageScope version 6.25 software (Aperio Technologies). The ImageScope algorithm for determining the intensity of membrane-specific staining was used to calculate the staining intensity and percent target label for each sample by digitally analyzing the color intensity. The output of stain intensities ranging from 0 to 3 correlated with conventional manual scoring methods (where 0 = negative and 3 = strong staining).

### Cell culture

Human OvCa cell lines (OVCAR-3 and CAOV-3) were obtained from the American Type Culture Collection (ATCC). The cells were cultured in RPMI 1640 (Mediatech, Inc.), supplemented with 10% fetal bovine serum (FBS, Sigma) at 37°C with 5% CO_2_. Prior to each experiment, cells were cultured for 24 hours in RPMI 1640 and 2% charcoal-striped FBS.

### Primer design

Human mRNA sequences for CCR9, MMP-1, MMP-2, MMP-3, MMP-7, MMP-8, MMP-9, MMP-10, MMP-11, MMP-13 and 18 S rRNA were obtained from National Center for Biotechnology Information (NCBI) Gen Bank database accession numbers were XM003251, MM002421, NM004530, NM002422, XM017384, NM002424, NM04994, NM002425, NM005940, NM002427, and X00686.1, respectively. These sequences were then used to design primers for reverse transcription polymerase chain reaction (RT-PCR) analysis, which generated amplicons of 162, 83, 95, 155, 169, 86, 79, 94, 107, 117, 176, and 149 bp in size for CCR9, MMP-1, MMP-2, MMP-3, MMP-7, MMP-8, MMP-9, MMP-10, MMP-11, MMP-12, MMP-13 mRNA and 18 S rRNA, respectively. Primers were designed using the Primer 3 software program from the Whitehead Institute at the Massachusetts Institute of Technology. Thermodynamic analysis of the primers was conducted using Primer PremierTM (Integrated DNA Technologies) and MIT Primer III. The resulting primer sets were compared against the entire human genome to confirm specificity and to ensure that the primers flanked mRNA splicing regions.

### RNA isolation and RT-PCR

Total RNA from OvCa cells was isolated using Tri-Reagent, according to manufacturer's protocols (Molecular Research Center). Potential genomic DNA contamination was removed from the samples by treatment with RNase-free DNase (Invitrogen) for 15 minutes at 37°C. RNA was precipitated and resuspended in RNA Secure (Ambion). cDNA was generated by reverse transcribing 1.5 μg of total RNA using iScript reagents (BioRad) according to manufacturer's protocol (BioRad). cDNA was amplified with specific primers for CCR9, MMP-1, MMP-2, MMP-3, MMP-7, MMP-8, MMP-9, MMP-10, MMP-11, MMP-13 and 18 S rRNA using SYBR Green polymerase chain reaction master mix reagents (BioRad). PCR-Ready cDNA from normal adult ovaries was obtained from Spring Bioscience. The number of copies (>5) of mRNA relative to 18S rRNA copies of these targets was evaluated by RT-PCR analysis using the BioRad Icycler and software. Hence, the number of copies for each target was calculated using a standard curve and data were normalized with copies of 18 S rRNA expressed in each sample. The results are presented as the number of copies of target per 10^6 ^copies of 18 S rRNA. Gene expression analysis experiments were repeated twice.

### Flow cytometry analysis of CCR9 surface expression

Phycoerythrin (PE)-conjugated mouse anti-human CCR9 (clone 112509) antibody and PE-conjugated mouse IgG2a immunoglobulin isotype control (clone 20102) was purchased from R&D Systems. OvCa cells were washed three times in phosphate buffered saline (PBS) [supplemented with 1% bovine serum albumin (BSA)] and treated with 1.0 μg of Fc Block (PharMingen) per 10^5 ^cells for 15 minutes at room temperature. Fc-blocked cells were stained with 1.0 μg of PE-conjugated mouse anti-human CCR9 or PE-conjugated mouse IgG2a isotype control antibody per 10^5 ^cells at 4°C for 1 hour. Subsequently, the cells were washed with 1.0 mL of fluorescence-activated cell-sorting (FACS) buffer (1% BSA in PBS) to remove unbound antibodies. Next, labeled cells were fixed in 500 μL of 2% paraformaldehyde solution, and 10^5 ^cells were analyzed by flow cytometry using a FACScan flow cytometer and CellQuest software (BD PharMingen).

### Migration and invasion assays

OvCa cell migration and Matrigel invasion chambers were obtained from Becton Dickinson Discovery Labware. Serum free carbonate-base Dulbecco's Modified Eagle's Medium (DMEM) was added to the bottom chamber (750 μL) and top chamber (500 μL) of the martigel inserts and allowed to hydrate for 2 hours at 37°C and 5% CO_2_. After hydration, media was gently aspirated from bottom and top chambers, while 100 ng/mL of CCL25 (PeproTech) or albumin (negative control) was prepared in RPMI supplemented with charcoal stripped FBS and 750 μl added to the bottom chamber. Next, 10^4 ^cancer cells in 500 μL of RPMI with or without 1 (g/mL of the mouse anti-human CCR9 (clone 112509, R&D Systems) or isotype control (clone 20102; R&D Systems) antibodies were added to the top chamber of the inserts and incubated overnight at 37°C with 5% CO_2_. After incubation, cells from the top chamber were removed using a cotton-tipped swab. Cells that migrated or invaded through and to the bottom surface of the inserts were fixed with 100% methanol for 2 minutes, stained for 2 minutes in 1% toluidine blue (Sigma) supplemented with 1% borax (Sigma) and rinsed twice with distilled water. The cells were counted by microscopy; migration and invasion studies were repeated three times.

### Active MMP detection

OvCa cells (10^5 ^per well) were seeded in 24-well plates and treated with 0 or 100 ng/mL of CCL25, 1 μg/mL of isotype control antibody or anti-CCR9 antibody, or culture media without cells. Conditioned media from the untreated and treated cells were collected for subsequent analysis of active MMP expression. Flurokine (R&D Systems) and Biotrak (GE healthcare) assay kits were used to quantify active collagenases, gelatinases, and stromelysins in the conditioned media, according to manufacturer's protocols.

### Statistics

CCR9 expression intensity by ovarian TMAs was tested for normality assumptions using the Shapiro-Wilk test and transformed to a logit scale. The general linear models (GLM) procedure was used to test the association of CCR9 expression and disease condition using SAS version 9.1.3 statistical analysis software. Results were declared significant at a α level of 0.001. The experimental data were compared using a two-tailed Student's *t *test and expressed as the mean ± SEM. The results were analyzed using the Stat view II program (Abacus Concepts, Inc.) and were labeled statistically significant if *p *values were < 0.01. When MMP levels were lower than the detectable limit of the assays, the values were recorded as one-half of the minimum detection limit for statistical analysis. Using the Cell Quest Software, the Kolmogorov-Smirnov (K-S) two-sample test was used to calculate the statistical significance of the CCR9 flow cytometry histograms.

## Results

### Expression of CCR9 by OvCa tissue

**Figure 1:**
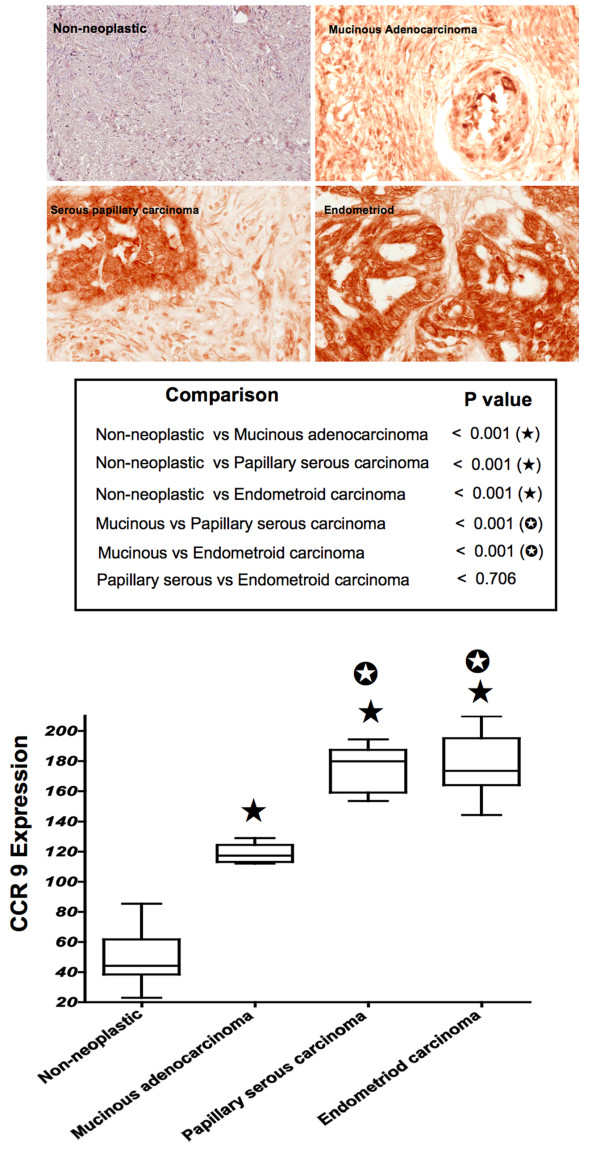
**CCR9 expressed by ovarian cancer tissue**. Ovarian cancer tissues from non- neoplastic (n = 8), mucinous adenocarcinoma (n = 5), serous papillary carcinoma (n = 10), and endometroid carcinoma (n = 11) were stained with isotype control or anti-CCR9 antibodies. Brown (DAB) color show CCR9 staining. An Aperio ScanScope CS system with a 40× objective captured digital images of each slide. Representative cases are indicated and immuno-intensities of CCR9 were quantified using image analysis Aperio ImageScope v.6.25 software. CCR9 expression by tissues were analyzed and presented by modified box plot. Lower, middle and upper lines, respectively, in the box represent the first quartile (Q1), Median (Q2) and third quartile (Q3). Upper (T) and lower (⊥) whiskers are represented by median ± 1.5 (Q3-Q1). Significant differences from non-neoplastic are indicated with a solid star whereas significant differences between mucinous adenocarcinoma and serous papillary as well as endometroid carcinomas are indicated with a white star in a black circle.

Ovarian TMAs consisting of non-neoplastic, mucinous adenocarcinoma, papillary serous carcinoma, and endometriod carcinoma tissues were evaluated for CCR9 expression. Positive staining was classified as 1 (missing or weak expression), 2 (medium expression), or 3 (high expression). In general, OvCa tissues significantly (*p *< 0.001) expressed CCR9 compared to non-neoplastic tissue, as did papillary serous and endometroid carcinomas compared to mucinous adenocarcinoma (Figure 1). The highest expression of CCR9 was observed in endometriod carcinoma followed by papillary serous carcinomas. While CCR9 expression by mucinous adenocarcinoma was lower than endometriod and papillary serous carcinomas, these OvCa cases significantly (*p *< 0.001) expressed CCR9 compared to non-neoplastic ovarian tissue.

### CCR9 expression by OvCa cell lines

**Figure 2:**
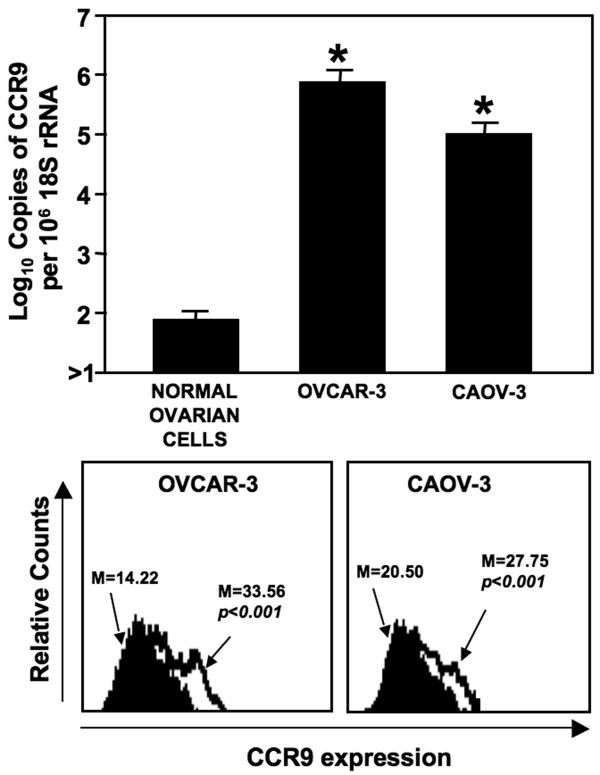
**CCR9 mRNA and cell surface protein expressed by ovarian cancer cells**. Total RNA was isolated from OVCAR-3 and CAOV-3 cell lines and normal primary ovary tissue. Quantitative RT-PCR analysis of CCR9 mRNA expression was performed in triplicate. The copies of transcripts are expressed relative to actual copies of 18 S rRNA ± SEM. OVCAR-3 and CAOV-3 cells were stained with PE-conjugated isotype control antibodies (solid histogram) or PE-conjugated anti-CCR9 monoclonal antibodies (open histogram) and quantified by flow cytometry. The mean fluorescent intensities of PE-positive cells are shown. Asterisk(s) indicate statistical significance (*p *< 0.01) between normal tissue and OvCa cells.

OvCa cell lines as well as non-neoplastic ovarian epithelial cells were evaluated for CCR9 mRNA and OVCAR-3 and CAOV-3 cell lines were characterized for CCR9 protein expression. CCR9 mRNA was significantly (*p *< 0.01) expressed by OVCAR-3 and CAOV-3 cell lines compared to normal ovarian epithelial cells (Figure 2). CCR9 surface protein expression was evaluated by flow cytometry. As with mRNA expression, OvCa cell lines significantly expressed CCR9 compared to controls. The mean fluorescent intensity of CCR9 expression for OVCAR-3 (M = 33.56) was significantly higher than CAOV-3 (M = 27.75).

### CCL25-induced migration and invasion of OvCa cell lines

**Figure 3:**
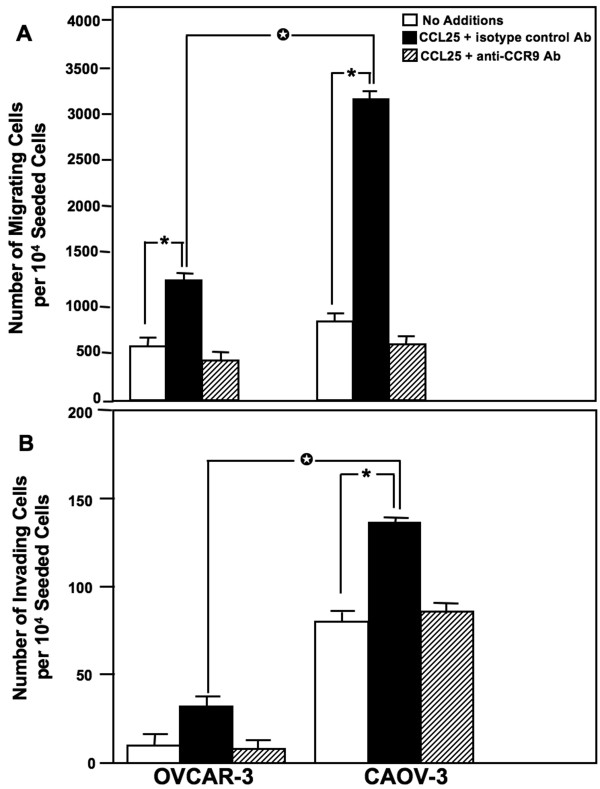
**CCR9-mediated ovarian cancer cell migration and invasion**. (A) OVCAR-3 and CAOV-3 cells were tested for their ability to migrate toward chemotactic gradients of CCL25. Cells were co-cultured with 1.0 μg/mL mouse anti-CCR9 antibody during migration assays using 100 ng/mL of CCL25. (B) OVCAR-3 and CAOV-3 cells were also tested for their ability to invade or translocate across Matrigel™ matrix in response to 100 ng/mL of CCL25. Cells were co-cultured with 1.0 μg/mL monoclonal antibodies against CCR9 during invasion assays using 100 ng/mL of CCL25. The number of cells ± SEM that migrated or invaded is shown with asterisk(s) that indicate significant differences (p < 0.01) between no additions and chemokine-induced cells.

OvCa cell lines were tested for CCL25-dependent migration and invasion. CAOV-3 and OVCAR-3 cells significantly migrated to CCL25, compared to media without CCL25 (Figure 3). This CCL25-dependent chemotaxis was neutralized by anti-CCR9 antibody treatment, but not by the isotype control antibody. These findings demonstrated the functional expression of CCR9 by OvCa cells, which migrate to CCL25. CAOV-3 and OVCAR-3 differentially invaded Matrigel in response to CCL25. CAOV-3, but not OVCAR-3, cell lines significantly invaded through Matrigel in response to CCL25. As with migration responses, CCL25-mediated invasion was CCR9-dependent since cell lines treated with anti-CCR9 antibody behaved like controls. Interestingly, the differences in cell invasion did not correlate with CCR9 expression, because OVCAR-3 cell lines expressed significantly more CCR9 than CAOV-3 cells.

### CCL25-induced MMP expression by OvCa cells

**Figure 4:**
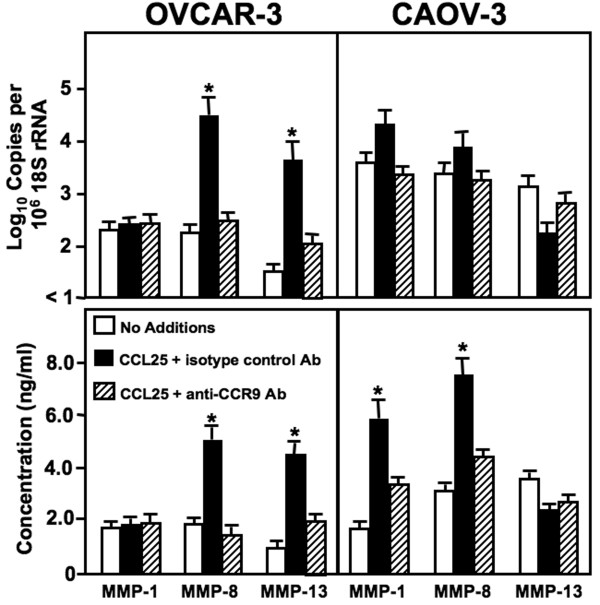
**CCL25-induced collagenase expression by ovarian cancer cells**. Cells were tested for their ability to express collagenases (MMP-1, MMP-8, and MMP-13) mRNA and protein. OVCAR-3 and CAOV-3 cells were cultured for 24 hours alone, with 100 ng/mL of CCL25, or CCL25 + 1 μg/mL of mouse anti-CCR9 antibody. Total RNA was isolated and quantitative RT-PCR analysis was performed for mRNA expression of collagenases (upper panel) and transcript copies are presented relative to actual copies of 18 S rRNA. Active collagenases were quantified by Fluorokine and Biotrak assays in conditioned media (lower panel). MMP expression below the detectable limit of the RT-PCR is designated as below detection (BD). Asterisk(s) indicate statistical differences (*p *< 0.01) between untreated and CCL25-treated OvCa cells.

To determine the mechanisms behind CCR9-dependent OvCa cell invasion and the enhanced ability of CAOV-3 cells compared to OVCAR-3 cell lines to invade Matrigel in response to CCL25, we quantified the expression of MMP mRNA and active protein. Both untreated and CCL25-treated OVCAR-3 and CAOV-3 cell lines expressed collagenases (MMP-1, -8, and -13) (Figure 4). Compared to untreated cells, CCL25-treated OVCAR-3 cells significantly expressed MMP-8 and MMP-13 mRNAs and active proteins. While CCL25 treatment of CAOV-3 cells did not affect collagenase mRNA expression, CCL25-treated CAOV-3 cells significantly expressed MMP-1 and -8 active protein, compared to untreated controls or CCL25-treated cells co-incubated with anti-CCR9 antibody. However, MMP-13 mRNA and active protein expression by CAOV-3 cells was not affected by CCL25 stimulation.

**Figure 5:**
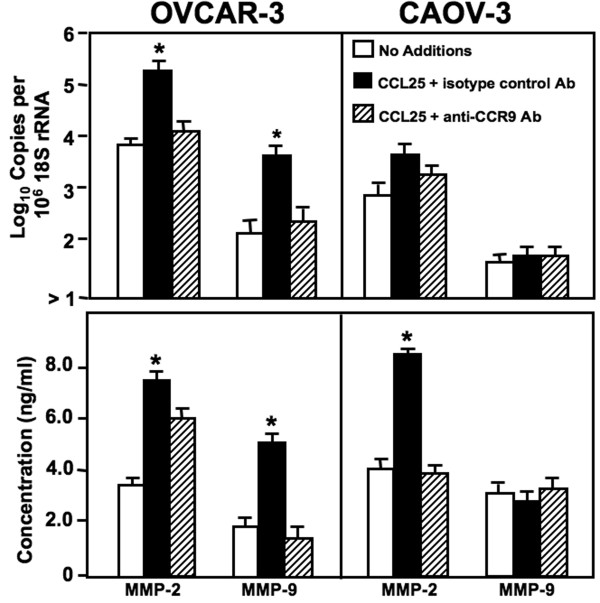
**CCL25-induced gelatinase expression by ovarian cancer cells**. Cells were tested for their ability to express gelatinases (MMP-2 and MMP-9) mRNA and protein. OVCAR-3 and CAOV-3 cells were cultured for 24 hours alone, with 100 ng/mL of CCL25, or 100 ng/mL of CCL25 + 1 μg/mL of monoclonal antibodies against CCR9. Total RNA was isolated and quantitative RT-PCR analysis was performed for mRNA expression of gelatinases (upper panel) and transcript copies are presented relative to actual copies of 18 S rRNA. Active gelatinases in conditioned media were quantified by Fluorokine and Biotrak assays (lower panel). MMP expression below the detectable limit of the RT-PCR is designated as below detection (BD). Asterisk(s) indicate statistical differences (*p *< 0.01) between untreated and CCL25-treated OvCa cells.

**Figure 6:**
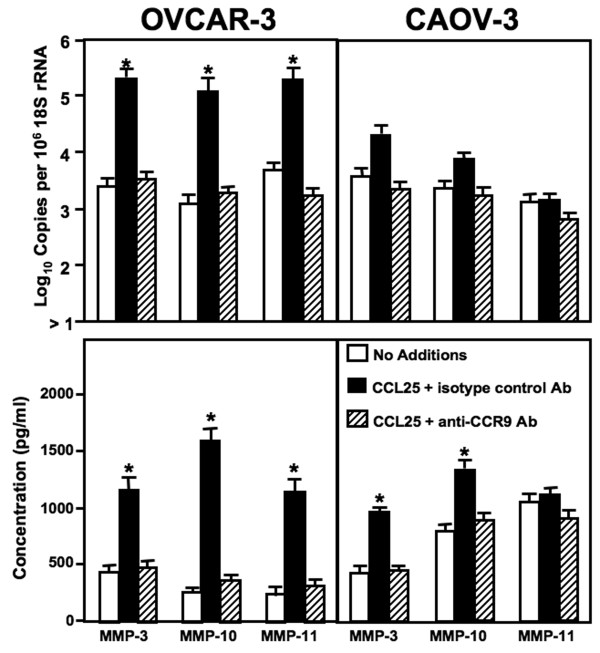
**CCL25-induced stromelysin expression by ovarian cancer cells**. Cells were tested for their ability to express stromelysins (MMP-3, MMP-10, and MMP-11) mRNA and protein. OVCAR-3 and CAOV-3 cells were cultured for 24 hours alone, with 100 ng/mL of CCL25, or 100 ng/mL of CCL25 + 1 μg/mL of monoclonal antibodies against CCR9. Total RNA was isolated and quantitative RT-PCR analysis was performed for mRNA expression of stromelysins (upper panel) and transcript copies are presented relative to actual copies of 18 S rRNA. Active stromelysins were quantified by Fluorokine and Biotrak assays in conditioned media (lower panel). MMP expression below the detectable limit of the RT-PCR is designated as below detection (BD). Asterisk(s) indicate statistical differences (*p *< 0.01) between untreated and CCL25-treated OvCa cells.

MMP-2 mRNA expression and active protein were expressed by all OvCa cell lines (Figure 5). Following CCL25 treatment, OVCAR-3 cells significantly expressed MMP-2 and -9 mRNA as well as active protein compared to untreated cells. This gelatinase production was abrogated by anti-CCR9 antibody. MMP-9 mRNA and active protein by CAOV-3 cells was marginal and not affected by CCL25. While CCL25 treatment did not induce MMP-2 mRNA expression, active MMP-2 protein was generated after CCL25 stimulation; this increase was inhibited by anti-CCR9 antibody. Untreated OVCAR-3 and CAOV-3 cells expressed mRNA and active protein of stromelysins (Figure 6). To this end, untreated CAOV-3 cells produced more MMP-10 and -11 mRNA and active protein than OVCAR-3 cells. CCL25 treatment of OVCAR-3 cells induced large increases in MMP-3, -10, and -11 mRNAs and active proteins, while CCL25 treatment of CAOV-3 cells resulted in selective yet significant increases in active MMP-3 and -10 secretion.

## Discussion

Late diagnosis and metastasis are major causes for the high mortality rate of OvCa [1]. Chemokines have been shown to play important roles in organ-specific homing of cancer cells to distant organs [12,20]. CCL25-CCR9 interactions are key to leukocyte homing to the small bowel [21,22], a common and fatal site of OvCa metastasis. In this regard, high levels of CCL25 in the gut mucosa and expression of CCR9 by OvCa cell lines suggest CCL25-CCR9 interactions might play a significant role in the mucosal homing of OvCa cells.

While metastasis of well-differentiated endometrioid adenocarcinoma are typically limited to the uterine body, mildly and poorly differentiated invasive endometroid adenocarcinomas have been associated with cervical invasion and distal metastasis [23]. Serous papillary carcinoma is an aggressive form of endometrial cancer that is likely to present with deep myometrial invasion and lymph vascular involvement. By the time most affected women are diagnosed, serous papillary carcinoma often spreads outside the uterus. Serous endometrial intra-epithelial carcinoma is a recently recognized entity with the same cytological features and p53 mutations as uterine serous carcinoma, with the former associated with stromal and/or myometrial invasion and extra-uterine metastasis. Interestingly, we found the highest expression of CCR9 in serous papillary and endometroid carcinomas in comparison to non-neoplastic and to a lesser degree in mucinous adenocarcinoma cases. Given the poor prognosis of serous papillary and endometroid carcinoma, our data supports the development of therapies that target the CCL25-CCR9 axis. Indeed, CCR9 blockade inhibited migration, MMP production, and invasion of OvCa cell lines.

CCL25-CCR9 interactions have been previously implicated in the progression of melanoma and prostate cancers [10,24]. Other studies concluded CCR9 is highly expressed by melanoma cells and all melanoma cells isolated from small intestine metastases [25]. Here we show for the first time that normal ovarian epithelial cells and non-neoplastic tissues express low levels of CCR9, while OvCa cell lines and mucinous adenocarcinoma, papillary serous carcinoma, and endometriod carcinoma tissues express high levels of CCR9. While ovulating ovaries express CCR9 and CCL25, which play important role during ovulation [26], we show that the expression of CCR9 mRNA by OvCa cell lines is significantly higher than levels expressed by normal adult ovarian epithelial cells. Each of the OvCa cell lines exhibited significantly higher CCL25-mediated migration and invasion, which was CCR9-dependent.

Among the numerous OvCa cell lines studied, CAOV-3 and OVCAR-3 have the highest incidence and average metastatic frequency [27]. CAOV-3 is an invasive human ovarian papillary carcinoma cell line. While the histological phenotype of the OVCAR-3 cell line is unknown, it was established from the malignant ascites of a patient with progressive adenocarcinoma of the ovary. OVCAR-3 xenografts produce either ascites or solid tumors in the peritoneal cavity [28], but similar grafts using the more invasive CAOV-3 cell line result in lung and other organ metastasis [27]. Neoplastic cells must penetrate the basement membrane and invade the interstitial stroma to initiate the metastatic process. To this end, many proteinases are capable of degrading extracellular matrix (ECM) components, but MMPs appear to be particularly important for matrix degradation [29,30] and cancer cell dissemination [31].

Collagenases (MMP-1, MMP-8 and MMP-13) initiate degradation of several naïve fibrillar collagens, including type-I, -II and -III. Higher expression of MMP-1 has been correlated with progression and poor survival in bladder cancer [32]. In most instances, increased expression of MMP-1 has a significant negative correlation with survival. Similarly, over production of MMP-8 has been shown to contribute to the invasive potential of OvCa [33]. Indeed, MMP-8 expression significantly correlated with ovarian tumor grade, tumor stage, and poor prognosis [34]. Even though CAOV-3 cells expressed slightly less CCR9 than OVCAR-3 cells, CCL25 treatment resulted in significant MMP-1 and -8 mRNA and active protein expression by CAOV-3 >> OVCAR-3 cell lines. On the other hand, MMP-13 is important for the degradation of type-I and -II collagens and its presence in ascites fluid has been used to identify patients at risk for early death from OvCa [7]. MMP-13 has been well documented in many aggressive cancers, but its expression is most often seen only in the invading front of tumors [35,36]. While OVCAR-3 cell supernatants expressed more MMP-13 in response to CCL25 treatment, the more invasive CAOV-3 cells did not produce MMP-13 mRNA or active protein in response to this CCR9 ligand. Possibly, MMP-13 is differentially expressed by OvCa cells and not crucial for ovarian tumor invasiveness *per se*.

Gelatinase-A and -B (MMP-2 and -9), also called type IV collagenases, degrade gelatin, collagen and other basement membrane components. High levels of MMP-2 and -9 have been associated with many diseases, including OvCa, and correlate with poor prognosis [37]. In an immunohistochemical study of malignant ovarian tissues, positive staining of MMP-2 was associated with poor survival [38]. In the present study, OVCAR-3 and CAOV-3 cell lines expressed MMP-2 mRNA and active protein. Importantly, CCL25 treatment led to a significant increase in MMP-2 expression by both OvCa cell lines. MMP-9 is frequently up regulated by cancer cells and has been shown to affect tumor metastasis and progression. CCL25 treatment induced an increase in MMP-9 expression by OVCAR-3, but not CAOV-3 cells. Perhaps, the lack of active MMP-9 expression by these cell lines is due to the production of high levels of tissue inhibitors of metalloproteinases (TIMPs). TIMPs are major regulators of matrix metalloproteinase activity. Specifically, TIMP-1 preferably binds and inactivates MMP-9 [39]. In this regard, high circulating TIMP-1 correlated to the aggressive phenotype and unfavorable prognosis of malignant neoplasias [39]. Therefore, it is possible that the CAOV-3 cell lines express elevated levels of TIMP-1, which would inhibit the generation of active MMP-9 protein.

Stromelysins (MMP-3, -10, and -11) are typically expressed by normal epithelial cells and degrade a variety of substrates, including type IV, V, IX AND X collagens, fibronectin, laminin, elastin, and proteoglycan core proteins. Many carcinomas express stromelysins; for example, MMP-3 and -10 produced by the head and neck carcinomas are higher than in normal-matched tissue [40]. In this study, MMP-3 and MMP-10 mRNA and protein were expressed at significantly higher levels by OVCAR-3 and CAOV-3 cell lines, after CCL25 treatment. CCR9 activation also led to an elevation of MMP-11 mRNA and active protein expression by OVCAR-3 cells. Indeed, MMP-11 (or stromelysin-3) expression is more frequently observed in malignant ovarian carcinomas than tumors with low malignant potential [41].

Our study shows that the OvCa cell lines, OVCAR-3 and CAOV-3, differentially expressed MMPs that are important for OvCa metastasis after CCL25 stimulation. While OVCAR-3 and CAOV-3 cell lines were both established from malignant ascites [42], these cell lines selectively migrated chamber inserts and invaded Matrigel in response to CCL25. For example, OVCAR-3, but not CAOV-3, cells poorly attached to host-tissue surfaces and express lamin receptor [43]. While these responses were CCR9-dependent, other factors no doubt contribute to their abilities to migrate and invade tissue extracellular matrix components.

## Conclusions

We provide the first evidence that OvCa cells express functional CCR9. The effect of CCL25 on MMP expression suggests that this chemokine plays a role in ovarian tumor cell invasion via MMP modulation. Our results, along with selective expression of CCL25 in the small bowel, support our hypothesis that OvCa cell migration and invasion are in part mediated by CCL25-CCR9 interactions. Additional studies will be necessary to evaluate the other possible cellular and molecular mechanisms mediated by CCL25 that support OvCa cell migration and invasion.

## Competing interests

The authors declare that they have no competing interests.

## Authors' contributions

ELJ conducted the experiments and drafted the manuscript. RS analyzed the data and assisted with manuscript preparation. CMJH, WEG, EEP, and SS assisted during the experiments and manuscripts preparation. JWL conceptualized, edited, and revised the manuscript. All authors have read and approved the final manuscript.
